# Azelaic acid alleviates UVB-induced photoaging in keratinocytes by restoring Smad-dependent TGF-β signaling

**DOI:** 10.3389/fmed.2026.1758702

**Published:** 2026-04-07

**Authors:** Wenbing Lai, Lurun Wang, Wei Tang, Yuze Liu

**Affiliations:** 1 Department of Dermatology, Taihe Hospital, Hubei University of Medicine, Shiyan, Hubei, China; 2 Hepatobiliary and Pancreatic Surgery Center, Taihe Hospital, Hubei University of Medicine, Shiyan, Hubei, China; 3 Quality Control Office, Taihe Hospital, Hubei University of Medicine, Shiyan, Hubei, China

**Keywords:** Azelaic acid, extracellular matrix, keratinocytes, photoaging, TGF-β/Smad signaling, UVB

## Abstract

**Background:**

Skin photoaging, primarily caused by chronic ultraviolet B (UVB) exposure, is characterized by cellular senescence and extracellular matrix (ECM) degradation. Suppression of transforming growth factor-β (TGF-β)/Smad signaling plays a critical role in UVB-induced collagen loss and impaired skin repair. Azelaic acid (AzA), a clinically established dermatological agent with antioxidant and anti-inflammatory properties, has been reported to maintain skin homeostasis; however, its mechanistic role in protecting against UVB-induced photoaging remains unclear.

**Objective:**

This study aimed to investigate whether AzA alleviates UVB-induced photoaging in keratinocytes and to determine whether its protective effects are mediated through the restoration of Smad-dependent TGF-β signaling.

**Methods:**

An in vitro UVB-induced photoaging model was established using HaCaT keratinocytes. Following UVB irradiation, cells were treated with AzA. Cell viability and cytotoxicity were evaluated using CCK-8 and LDH assays, respectively. Cellular senescence was assessed by senescence-associated β-galactosidase staining. ECM-related markers were measured by ELISA. TGF-β/Smad signaling activity was analyzed by Western blotting, quantitative real-time PCR, and immunofluorescence. The selective TGF-β/Smad inhibitor SB431542 was used to verify pathway dependence. A keratinocyte–fibroblast conditioned medium model was also employed to assess the paracrine regulation of fibroblast ECM metabolism.

**Results:**

AzA significantly attenuated UVB-induced cellular senescence and partially restored cell viability in HaCaT cells. It significantly increased pro-collagen I levels and reduced MMP-1 levels, indicating the restoration of ECM-related protein balance. Mechanistically, AzA reversed UVB-induced suppression of TGF-β/Smad signaling by enhancing Smad2/3 phosphorylation, decreasing Smad7 expression, promoting the nuclear translocation of phosphorylated Smad2/3, and restoring Smad-dependent transcription. Pharmacological inhibition of TGF-β/Smad signaling largely abrogated these protective effects. Conditioned medium from AzA-treated keratinocytes also improved ECM-related responses in fibroblasts in a Smad-dependent manner.

**Conclusion:**

AzA alleviates UVB-induced photoaging by restoring Smad-dependent TGF-β signaling, preserving ECM homeostasis, and modulating epidermal–dermal crosstalk in vitro. These findings provide mechanistic support for the potential application of AzA in anti-photoaging strategies.

## Introduction

Skin photoaging is one of the most visible manifestations of extrinsic aging and is mainly caused by chronic exposure to ultraviolet B (UVB) radiation (280–320 nm) (1). It is characterized by wrinkles, dryness, loss of elasticity, pigmentation abnormalities, and impaired barrier function, which not only affect skin appearance but also increase susceptibility to inflammation and skin tumorigenesis (2, 3). Epidemiological studies estimate that more than 80% of visible facial aging is attributable to UV radiation and that the prevalence of clinical signs of photoaging increases with age, especially in chronically UV-exposed areas of the skin, such as the face and forearms (4, 5). Therefore, chronic UV exposure represents a major public health concern, contributing substantially to the global burden of dermatological disorders.

At the molecular level, UVB irradiation primarily affects epidermal cells and induces excessive production of reactive oxygen species (ROS), leading to DNA damage, lipid peroxidation, and mitochondrial dysfunction (6, 7).

UVB-induced oxidative stress activates multiple intracellular signaling pathways implicated in photoaging, including the MAPK/AP-1 and NF-κB pathways, which promote the transcription of matrix metalloproteinases (MMPs), such as MMP-1 and MMP-3, thereby accelerating the degradation of type I and III collagen in the dermal extracellular matrix (ECM) (8, 9). These signaling cascades act in a coordinated manner to drive inflammation, ECM remodeling, and progressive skin aging, highlighting the complexity of UVB-induced photoaging at the molecular level.

In parallel, UVB exposure markedly suppresses the transforming growth factor-β (TGF-β)/Smad signaling pathway, which plays a central role in collagen synthesis, ECM homeostasis, and tissue repair in the skin (10). Reduced phosphorylation of Smad2/3 and upregulation of the inhibitory Smad7 impair TGF-β signaling, resulting in diminished collagen production, ECM disorganization, and accelerated photoaging (11). Notably, MAPK/AP-1 and NF-κB signaling have been reported to functionally antagonize TGF-β/Smad activity, thereby further exacerbating UVB-induced ECM degradation. Restoration of TGF-β/Smad signaling within this broader photoaging-related signaling network is therefore considered a critical strategy for maintaining ECM integrity and promoting skin repair. In human skin, TGF-β/Smad signaling operates in both epidermal keratinocytes and dermal fibroblasts; however, collagen synthesis and ECM production are predominantly executed by dermal fibroblasts (12). Keratinocytes, as the primary targets of UVB irradiation, play an upstream regulatory role in epidermal–dermal communication (13). Accumulating evidence indicates that UVB-induced alterations in keratinocyte signaling can influence fibroblast-mediated ECM remodeling through paracrine mechanisms (6, 14). Disruption of TGF-β/Smad signaling in keratinocytes may, therefore, impair the secretion of soluble mediators that regulate fibroblast collagen synthesis and MMP expression. Accordingly, restoration of epidermal Smad signaling could indirectly contribute to dermal ECM preservation during photoaging.

From a clinical and translational perspective, skin photoaging is increasingly recognized not merely as cumulative structural damage but as a chronic failure of endogenous repair and regeneration processes, including impaired ECM renewal and disruption of skin-barrier homeostasis. In this context, studies on chronic skin injury and repair have emphasized that sustained impairment of coordinated cellular responses and tissue homeostasis represents a common pathological denominator across diverse skin conditions driven by long-term environmental stress. These conceptual insights highlight the broader importance of endogenous repair mechanisms in maintaining skin integrity and are highly relevant to the pathophysiology of UVB-induced photoaging (15).

Azelaic acid (AzA), an endogenous nine-carbon saturated dicarboxylic acid present in grains, barley, and yeast-derived preparations (16), has long been used in clinical dermatology. AzA exhibits multiple biological activities, such as antioxidant, anti-inflammatory, antimicrobial, and keratinocyte-modulating effects (17). Clinically, it has been widely applied as a topical agent for acne vulgaris, rosacea, and hyperpigmentation due to its ability to normalize keratinization, inhibit tyrosinase activity, and scavenge free radicals (18). Emerging evidence further suggests that AzA contributes to the maintenance of skin homeostasis by improving epidermal function and protecting against oxidative stress, underscoring its translational relevance in dermatological applications (19). However, the molecular mechanisms by which AzA modulates UVB-induced skin photoaging, particularly regarding ECM regulation and repair-associated signaling pathways, remain incompletely understood.

Given the pivotal role of TGF-β/Smad signaling in ECM maintenance and tissue repair, as well as its functional interaction with other photoaging-related pathways, it was hypothesized that AzA exerts anti-photoaging effects by restoring Smad-dependent TGF-β signaling within the broader UVB-induced stress-response network. To test this hypothesis, a UVB-induced photoaging model was established using HaCaT keratinocytes, and the effects of AzA on cellular senescence, ECM metabolism, and TGF-β/Smad pathway activity were systematically evaluated. Furthermore, the selective TGF-β/Smad inhibitor SB431542 was employed to verify pathway dependence.

## Materials and methods

### Cell culture

The immortalized human keratinocyte cell line HaCaT (Cat. No. GNHu64, Cell Bank of the Chinese Academy of Sciences, Shanghai, China) and human dermal fibroblasts (HDFs) (Cat. No. GNHu12, Cell Bank of the Chinese Academy of Sciences, Shanghai, China) were used in this study. Cells were maintained in Dulbecco’s Modified Eagle Medium (DMEM) supplemented with 10% fetal bovine serum (FBS) and 1% penicillin–streptomycin solution and incubated at 37 °C in a humidified atmosphere containing 5% CO_2_.

### UVB irradiation

Cells were seeded in six-well plates and washed twice with phosphate-buffered saline (PBS) to remove serum prior to irradiation. UVB irradiation was performed using a UVB lamp (TL 20 W/01 RS SLV/25, peak emission at 315 nm, Philips, Amsterdam, Netherlands). The irradiation intensity was measured using a UV radiometer (VLX-3W, Vilber Lourmat, Marne-la-Vallée, France), and UVB exposure was controlled by energy output to deliver a final dose of 30 mJ/cm^2^ (14, 20, 21). Immediately after irradiation, PBS was replaced with fresh complete culture medium containing the indicated concentrations of AzA. Cells were then incubated for 24 h before subsequent analyses.

### Reagents and treatments

AzA (purity ≥ 99%, 123-99-9, Aladdin, Shanghai, China) was dissolved in sterile dimethyl sulfoxide (DMSO) and diluted in DMEM to final concentrations of 5, 10, 20, 30, and 40 mM (22). The final DMSO concentration in all treatments was maintained below 0.1%, which did not affect cell viability. The TGF-β/Smad signaling inhibitor SB431542 (HY-10431, MedChemExpress, Shanghai, China) was prepared as a 10 mM DMSO stock and then diluted down to a 10 μM working concentration, as previously reported (23). Cells were placed into the following predefined experimental groups: Control: untreated cells; UVB: 30 mJ/cm^2^ (UVB irradiation only); UVB + AzA (5, 10, 20 mM): cells exposed to UVB and treated with different concentrations of AzA; UVB + SB431542: UVB-irradiated cells treated with 10 μM SB431542; UVB + AzA + SB431542: co-treatment to assess pathway dependence. All treatments were performed for 24 h following UVB irradiation.

### Cell viability assay (CCK-8)

Cell viability was evaluated using a Cell Counting Kit-8 (CCK-8; C0037, Guangzhou Yujia Biotechnology, China) according to the manufacturer’s instructions. HaCaT cells were seeded at a density of 1 × 10^4^ cells per well in 96-well plates and allowed to attach overnight. Following UVB irradiation, cells were treated with AzA for 24 h.

Subsequently, 10 μL of CCK-8 solution was added to each well and incubated at 37 °C for 2 h. Absorbance was measured at 450 nm using a Multiskan SkyHigh Microplate Spectrophotometer (Thermo Fisher Scientific, Waltham, MA, USA). Blank wells containing culture medium and CCK-8 reagent without cells were included to correct for background absorbance. Cell viability was calculated relative to untreated control cells.

### LDH release assay

Cytotoxicity was further evaluated using a lactate dehydrogenase (LDH) cytotoxicity assay kit (C0016, Beyotime Biotechnology, Shanghai, China) according to the manufacturer’s instructions. Briefly, cell culture supernatants were collected after the indicated treatments and centrifuged at 1,000×*g* for 5 min to remove cellular debris. An aliquot of each supernatant was transferred to a new 96-well plate and incubated with the LDH reaction mixture at room temperature for 30 min in the dark.

Absorbance was measured at 450 nm using a Multiskan SkyHigh Microplate Spectrophotometer (Thermo Fisher Scientific, Waltham, MA, USA). LDH release was normalized to the control group and expressed as relative cytotoxicity.

### Senescence-associated β-galactosidase (SA-β-gal) staining

Senescence was evaluated via senescence-associated β-galactosidase (SA-β-gal) staining according to established methods (24) using a commercial kit (C1342264-100T, Aladdin, Shanghai, China). Cells subjected to UVB and AzA were washed, fixed for 15 min in 4% paraformaldehyde, and incubated at 37 °C overnight in CO_2_-free staining reagent (pH 6.0). Senescent cells displaying blue staining were visualized under a light microscope (DMI6000 B, Leica DMi8, Germany). For quantitative analysis, at least five random fields per well were captured. The percentage of SA-β-gal-positive cells was calculated as the number of blue-stained cells divided by the total number of cells in the same field ×100% using ImageJ software.

### Enzyme-linked immunosorbent assay (ELISA)

Pro-collagen I (97044ES48, Yeasen, Shanghai, China) and MMP-1 (JL10180-48T, Jianglai, Shanghai, China) levels were quantified in cell culture supernatants using commercial sandwich ELISA kits according to the manufacturers’ protocols. Supernatants were collected 24 h after UVB irradiation and AzA treatment (HaCaT cells) or from HDFs following incubation with conditioned media and then centrifuged at 1,000×*g* for 10 min at 4 °C.

Standard curves were generated using kit-provided standards, and concentrations were calculated accordingly. Detection was performed using an HRP–TMB colorimetric system, and absorbance was measured at 450 nm with a Multiskan SkyHigh microplate reader (Thermo Fisher Scientific, Waltham, MA, USA). Protein levels were normalized to total cellular protein.

### Immunofluorescence staining

Cells grown on coverslips were fixed with 4% paraformaldehyde, permeabilized with 0.1% Triton X-100, and blocked with 5% bovine serum albumin. Cells were incubated overnight at 4 °C with a primary antibody against phosphorylated Smad2/3 (p-Smad2/3; ab272332, Abcam, Cambridge, UK), followed by incubation with an appropriate fluorophore-conjugated secondary antibody (Alexa Fluor 488–conjugated goat anti-rabbit IgG, ab150077, Abcam). Nuclei were counterstained with 4′,6-diamidino-2-phenylindole (DAPI; C0065, Solarbio, China). Images were acquired using a fluorescence microscope, and nuclear-to-cytoplasmic fluorescence ratios were quantified using ImageJ software.

### Quantitative real-time PCR (qPCR)

Total RNA was extracted from HaCaT cells or HDFs using TRIzol reagent (Cat. No. 15596026, Invitrogen, USA) according to the manufacturer’s instructions. RNA concentration and purity were assessed using a NanoDrop spectrophotometer. Complementary DNA (cDNA) was synthesized from 1 μg of total RNA using a reverse-transcription kit (PrimeScript™ RT reagent Kit, Cat. No. RR037A, Takara, Japan).

Quantitative real-time PCR was performed using SYBR Green PCR Master Mix (TB Green® Premix Ex Taq™, Cat. No. RR420A, Takara, Japan) on a real-time PCR detection system. The amplification conditions were as follows: initial denaturation at 95 °C for 30 s, followed by 40 cycles of denaturation at 95 °C for 5 s and annealing/extension at 60 °C for 30 s. Gene expression levels of SERPINE1, CTGF, COL1A1, and COL1A2 were analyzed, with GAPDH used as the internal reference gene. Relative mRNA expression levels were calculated using the 2^−ΔΔCt^ method. Primer sequences are listed in Supplementary Table S1.

### Western blot analysis

Western blot analysis was performed according to established protocols (25). Total protein was extracted using RIPA lysis buffer (P0013C, Beyotime, China) supplemented with protease inhibitors. Cells were incubated on ice for 30 min and then centrifuged at high speed at 4 °C to remove insoluble debris. Protein concentrations were determined using a BCA Protein Assay Kit (P0012S, Guangzhou Yujia Biotechnology). Equal amounts of protein (30 μg per lane) were separated by 10% SDS-PAGE and subsequently transferred onto PVDF membranes (Millipore, USA).

The membranes were blocked with 5% skim milk for 1 h at room temperature and then incubated overnight at 4 °C with the following primary antibodies: phosphorylated Smad2/3 (p-Smad2/3; ab272332, Abcam), Smad2/3 (ab202445, Abcam), Smad7 (ab216428, Abcam), and β-actin (ab8226, Abcam). After washing with TBS-T, membranes were incubated with appropriate HRP-conjugated secondary antibodies for 1 h at room temperature. Protein bands were visualized using the SuperSignal™ West Pico PLUS chemiluminescent substrate (34580, Thermo Fisher Scientific, Waltham, MA, USA) and captured using a Bio-Rad ChemiDoc imaging system. Band intensities were quantified using ImageJ software (NIH, USA), with β-actin used as the loading control.

### Statistical analysis

All assays were performed in a minimum of three biological replicates, and values are shown as mean ± SD. Biological replicates represent independent batches of cell cultures. For Western blot analyses, experiments were repeated using independently prepared protein lysates. Statistical analyses were conducted using IBM SPSS Statistics, version 23.0 (IBM Corp., Armonk, NY, USA). Multiple group comparisons were carried out using one-way ANOVA followed by Tukey’s *post-hoc* test. A *p*-value of < 0.05 was considered significant, with significance denoted as *p* < 0.05, *p* < 0.01, and *p* < 0.001.

## Results

### AzA exhibited no significant cytotoxicity at concentrations up to 20 mM in HaCaT cells

**Figure 1:**
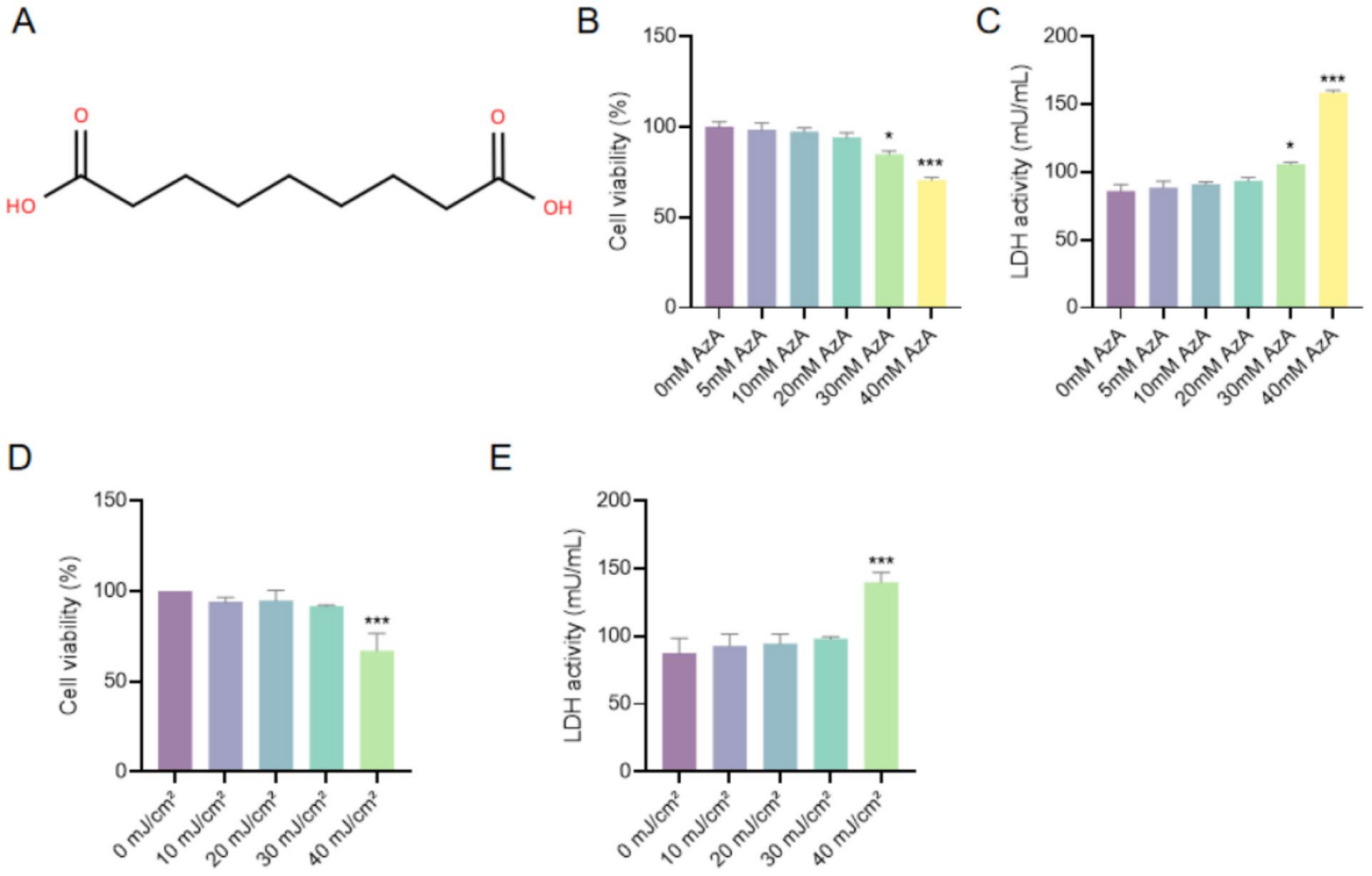
Chemical structure of AzA and determination of AzA and UVB treatment conditions in HaCaT cells. **(A)** Chemical structure of AzA. **(B)** Cell viability of HaCaT cells treated with different concentrations of AzA (5–40 mM) for 24 h, measured by the CCK-8 assay. **(C)** Cytotoxicity assessed by lactate dehydrogenase (LDH) release in HaCaT cells after AzA treatment for 24 h. **(D)** Cell viability of HaCaT cells exposed to increasing UVB doses (0–40 mJ/cm^2^), assessed by the CCK-8 assay. **(E)** LDH release in HaCaT cells following UVB irradiation at the indicated doses. Data are presented as mean ± SD from three independent experiments. Statistical analysis was performed using one-way ANOVA followed by Tukey’s *post-hoc* test. For panels **(B,C)**, *p < 0.05 and ***p < 0.001 vs. the 0 mM AzA group; for panels **(D,E)**, *p < 0.05 and ***p < 0.001 vs. the 0 mJ/cm^2^ UVB group.

As shown in Figure 1A, AzA is a linear saturated dicarboxylic acid containing nine carbon atoms with two terminal carboxyl groups. To determine the appropriate concentration range of AzA for subsequent experiments, HaCaT cells were treated with increasing concentrations of AzA (5–40 mM) for 24 h, and cell viability and cytotoxicity were evaluated.

The CCK-8 assay showed that AzA at 5, 10, and 20 mM did not significantly affect HaCaT cell viability, whereas AzA at 30 mM and 40 mM significantly reduced cell viability (*p* < 0.05 and *p* < 0.001, respectively; Figure 1B). In parallel, LDH release analysis indicated no significant change at AzA concentrations of 5–20 mM, whereas LDH activity was significantly increased at 30 mM and 40 mM (*p* < 0.05 and *p* < 0.001, respectively; Figure 1C), suggesting cytotoxicity at higher AzA concentrations.

To establish a stable UVB-induced photoaging model, HaCaT cells were exposed to different UVB doses (0, 10, 20, 30, and 40 mJ/cm^2^). UVB irradiation caused a dose-dependent decrease in cell viability, with a significant reduction observed at 40 mJ/cm^2^ (*p* < 0.001; Figure 1D). Consistently, LDH release increased with escalating UVB doses, with a significant elevation at 40 mJ/cm^2^ (*p* < 0.001; Figure 1E). Based on these results, a UVB dose of 30 mJ/cm^2^ and AzA concentrations of 5, 10, and 20 mM were selected for subsequent experiments.

### AzA attenuated UVB-induced cellular senescence and ECM degradation in HaCaT cells

**Figure 2:**
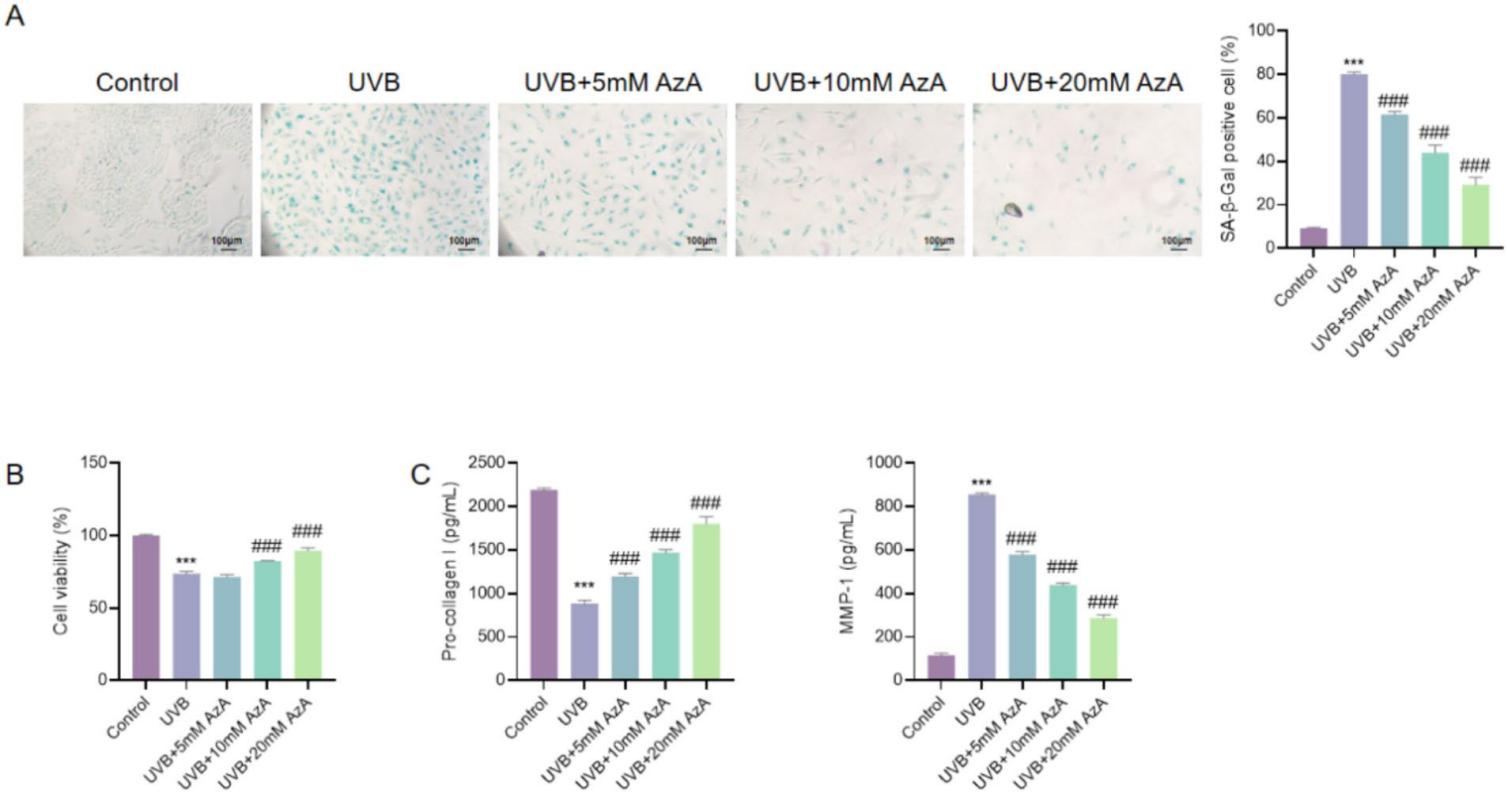
Effects of AzA on UVB-induced photoaging in HaCaT cells. **(A)** Representative SA-β-gal staining images and quantification of senescent cells (scale bar = 100 μm). **(B)** Cell viability analysis by CCK-8 assay. **(C)** Pro-collagen I levels detected by ELISA. **(D)** MMP-1 levels detected by ELISA. Data are presented as mean ± SD from three independent experiments. Statistical analysis was performed using one-way ANOVA followed by Tukey’s *post-hoc* test. ***p < 0.001 vs. the control group; ###*p* < 0.001 *vs.* the UVB group.

To assess whether AzA mitigates UVB-induced photoaging in keratinocytes, HaCaT cells were exposed to 30 mJ/cm^2^ UVB irradiation and subsequently treated with AzA at concentrations of 5–20 mM. As shown in Figure 2A, UVB irradiation markedly increased the proportion of SA-β-gal-positive cells compared with non-irradiated cells (*p* < 0.001), indicating pronounced cellular senescence. Compared with UVB-irradiated cells, AzA treatment significantly reduced the percentage of SA-β-gal-positive cells in a dose-dependent manner, with the greatest suppression observed at 20 mM (*p* < 0.001).

Consistently, the CCK-8 assay demonstrated that UVB irradiation significantly reduced HaCaT cell viability relative to non-irradiated cells (*p* < 0.001), whereas AzA treatment following UVB exposure progressively restored cell viability in a dose-dependent manner (*p* < 0.001) (Figure 2B).

In addition, ELISA analysis of HaCaT culture supernatants revealed that UVB irradiation led to a significant decrease in pro-collagen I levels and a concomitant increase in MMP-1 levels compared with non-irradiated cells (*p* < 0.001), indicating disruption of ECM-related protein balance. In contrast, AzA treatment after UVB exposure significantly increased pro-collagen I levels and reduced MMP-1 levels in HaCaT culture supernatants compared with UVB-irradiated cells (*p* < 0.001) (Figures 2C,D).

### AzA restored TGF-β/Smad signaling activity in UVB-irradiated HaCaT cells

**Figure 3:**
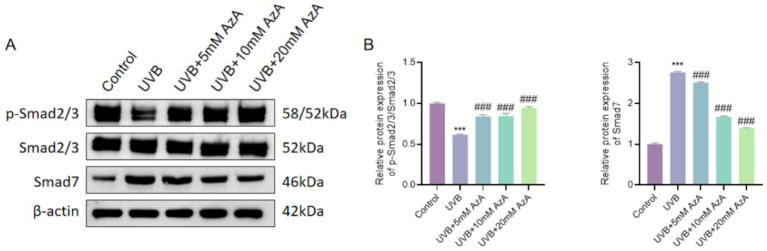
Effects of AzA on TGF-β/Smad signaling in UVB-irradiated HaCaT cells. **(A)** Representative western blot images showing the protein expression of p-Smad2/3, Smad2/3, and Smad7. **(B)** Quantitative analysis of p-Smad2/3 relative to Smad2/3. **(C)** Quantitative analysis of Smad7 protein expression. Data are presented as mean ± SD from three independent experiments. Statistical analysis was performed using one-way ANOVA followed by Tukey’s *post-hoc* test. ***p < 0.001 vs. the control group; ###*p* < 0.001 vs. the UVB group.

To explore whether AzA’s effects were mediated through the regulation of the TGF-β/Smad pathway, the protein expression levels of Smad2/3, phosphorylated Smad2/3 (p-Smad2/3), and Smad7 were examined by Western blotting. As shown in Figure 3A, UVB irradiation markedly reduced p-Smad2/3 levels and increased Smad7 expression compared with non-irradiated cells (*p* < 0.001), indicating suppression of TGF-β/Smad signaling.

Following UVB exposure, treatment with AzA significantly reversed these alterations in a dose-dependent manner. Compared with UVB-irradiated cells, AzA treatment at concentrations of 5–20 mM progressively increased p-Smad2/3 levels and decreased Smad7 expression (*p* < 0.001), while total Smad2/3 protein levels remained largely unchanged (Figures 3B,C). These results indicate that AzA functionally restores Smad-dependent TGF-β signaling suppressed during UVB-induced photoaging, thereby contributing to its protective effects in keratinocytes.

### Inhibition of the TGF-β/Smad pathway abrogated the protective effects of AzA against UVB-induced photoaging

To investigate whether the protective effects of AzA against UVB-induced photoaging depend on TGF-β/Smad signaling, HaCaT cells were treated with the selective TGF-β/Smad pathway inhibitor SB431542 alone or in combination with AzA under UVB irradiation.

**Figure 4:**
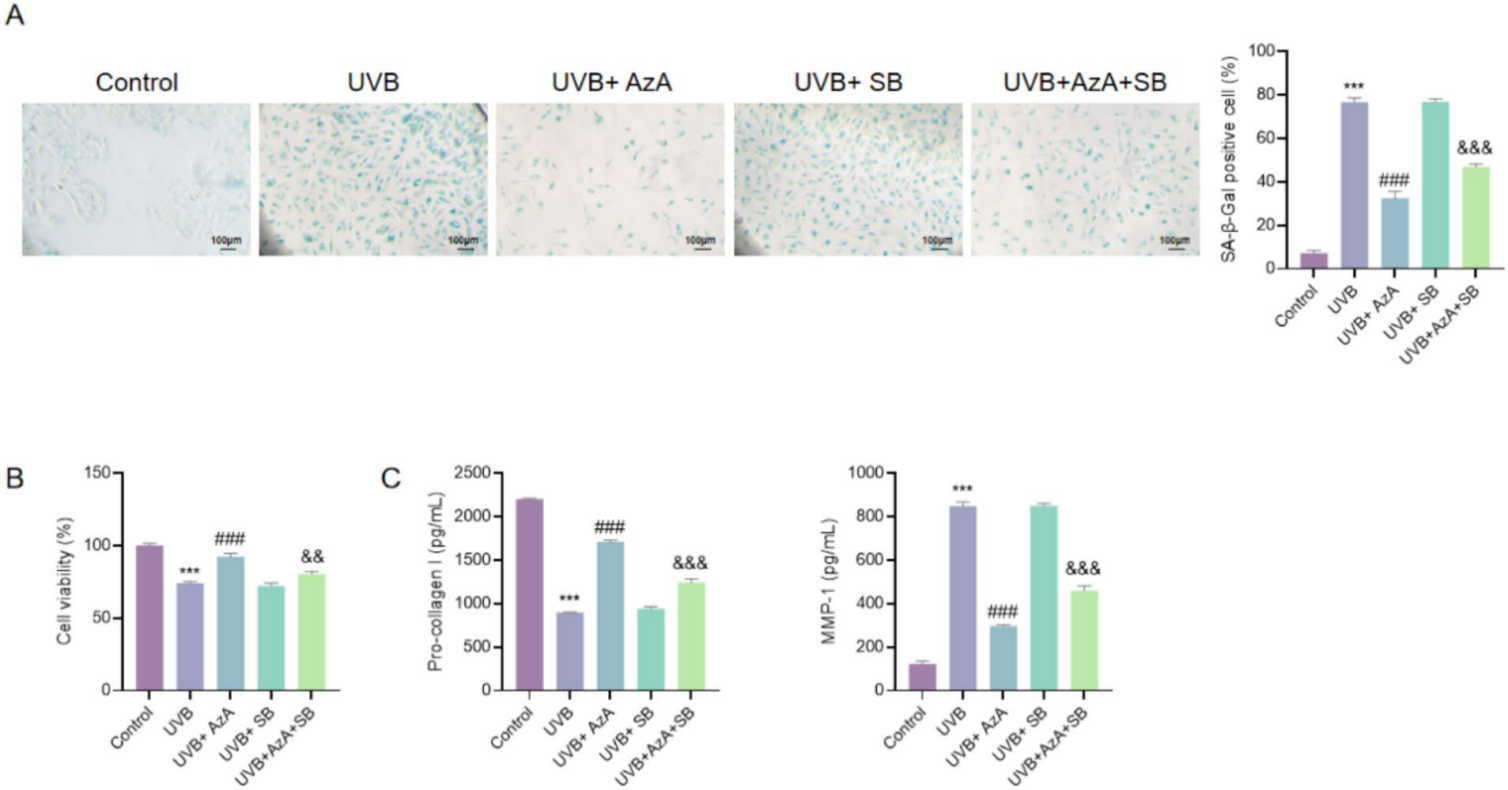
Inhibition of the TGF-β/Smad pathway attenuates the protective effects of AzA on UVB-induced photoaging. **(A)** Representative SA-β-gal staining images and quantification of senescent cells (scale bar = 100 μm). **(B)** Cell viability analysis by CCK-8 assay. **(C)** Pro-collagen I levels detected by ELISA. **(D)** MMP-1 levels detected by ELISA. Data are presented as mean ± SD from three independent experiments. Statistical analysis was performed using one-way ANOVA followed by Tukey’s *post-hoc* test. ***p < 0.001 vs. the control group; ###*p* < 0.001 vs. the UVB group; &&*p* < 0.01 and &&&*p* < 0.001 vs. the UVB + AzA group.

As shown in Figure 4A, UVB irradiation markedly increased the proportion of SA-β-gal-positive cells compared with non-irradiated cells (*p* < 0.001). AzA treatment following UVB exposure significantly reduced cellular senescence, whereas treatment with SB431542 alone did not attenuate UVB-induced senescence. Notably, co-treatment with SB431542 largely abolished the anti-senescence effect of AzA, resulting in a significant increase in SA-β-gal-positive cells compared with AzA treatment alone under UVB conditions (*p* < 0.001).

Consistently, the CCK-8 assay demonstrated that AzA effectively restored cell viability following UVB irradiation. When SB431542 was co-administered with AzA, the AzA-mediated recovery of cell viability was significantly suppressed, resulting in lower viability compared with AzA treatment alone (*p* < 0.01) (Figure 4B).

ELISA analysis further demonstrated that, compared with UVB-irradiated cells, AzA treatment markedly restored pro-collagen I levels and reduced MMP-1 levels in HaCaT culture supernatants (*p* < 0.001). Co-treatment with SB431542 largely abolished the protective effects of AzA, resulting in reduced pro-collagen I levels and increased MMP-1 expression compared with AzA-treated UVB-irradiated cells (*p* < 0.001) (Figures 4C,D).

### Inhibition of the TGF-β/Smad pathway reversed the protective effects of AzA in UVB-irradiated HaCaT cells

**Figure 5:**
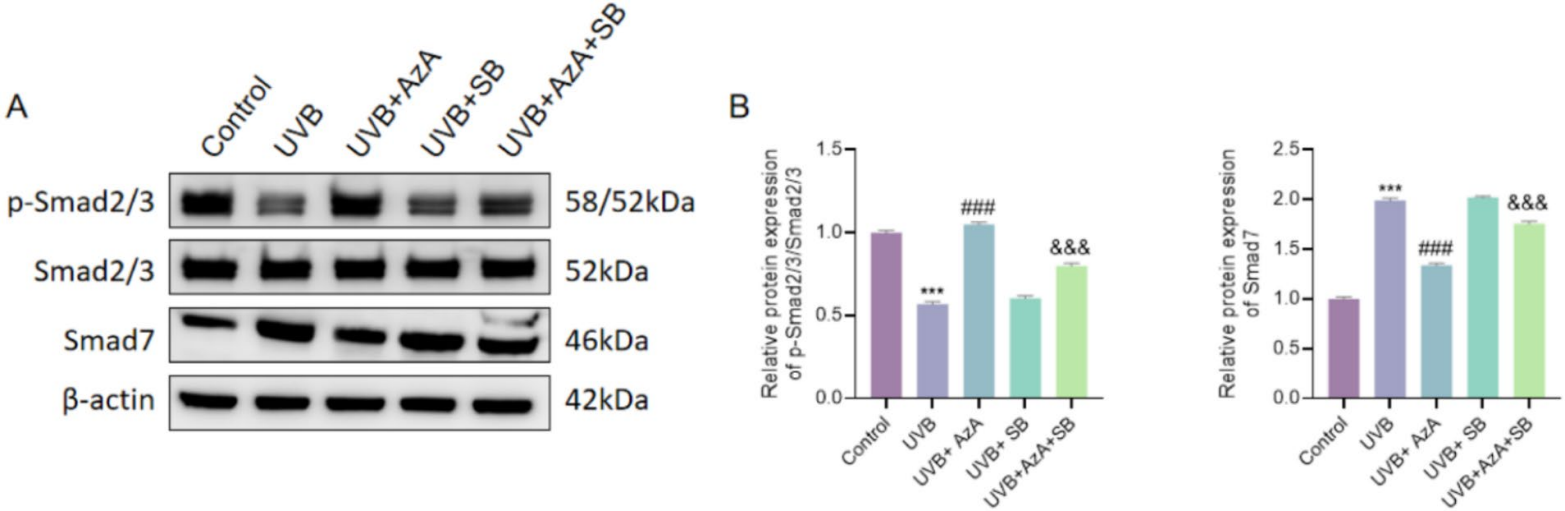
Inhibition of the TGF-β/Smad pathway reverses the protective effects of AzA in UVB-irradiated HaCaT cells. **(A)** Representative western blot images showing the protein expression of p-Smad2/3, Smad2/3, and Smad7. **(B)** Quantitative analysis of p-Smad2/3 relative to Smad2/3. **(C)** Quantitative analysis of Smad7 protein expression. Data are presented as mean ± SD from three independent experiments. Statistical analysis was performed using one-way ANOVA followed by Tukey’s *post-hoc* test. ***p < 0.001 vs. the control group; ###*p* < 0.001 vs. the UVB group; &&&*p* < 0.001 vs. the UVB + AzA group.

To examine whether activation of the TGF-β/Smad pathway underlies the anti-photoaging effects of AzA, the selective pathway inhibitor SB431542 was applied to UVB-irradiated HaCaT cells. As shown in Figure 5, UVB irradiation markedly reduced Smad2/3 phosphorylation and increased Smad7 expression compared with non-irradiated cells (*p* < 0.001). Treatment with AzA following UVB exposure significantly counteracted these alterations, resulting in increased p-Smad2/3 levels and decreased Smad7 expression.

In contrast, when SB431542 was co-administered with AzA following UVB irradiation, the AzA-induced increase in p-Smad2/3 and decrease in Smad7 expression were significantly attenuated, resulting in lower p-Smad2/3 levels and higher Smad7 expression compared with AzA treatment alone (*p* < 0.001). Together, these results demonstrate that blockade of TGF-β/Smad signaling effectively abrogates the molecular actions of AzA on Smad2/3 activation and Smad7 suppression, highlighting a critical role for this pathway in mediating the anti-photoaging effects of AzA in keratinocytes.

### AzA promotes p-Smad2/3 nuclear translocation and Smad-dependent transcription

**Figure 6:**
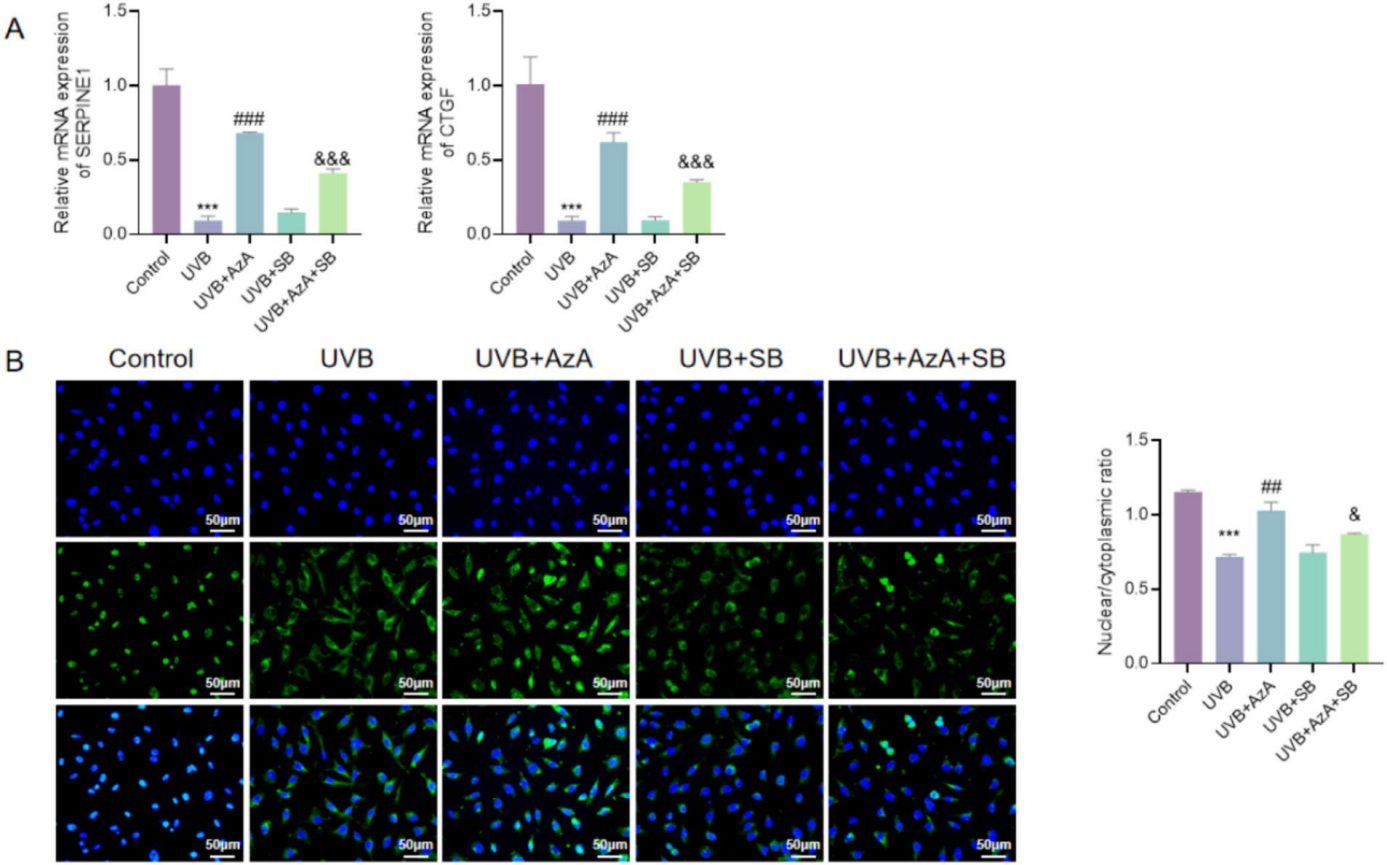
AzA promotes Smad-dependent transcription and promotes p-Smad2/3 nuclear translocation in UVB-irradiated HaCaT cells. **(A)** Relative mRNA expression levels of SERPINE1 in HaCaT cells under the indicated treatments, determined by quantitative real-time PCR. **(B)** Relative mRNA expression levels of CTGF in HaCaT cells under the indicated treatments, determined by quantitative real-time PCR. **(C)** Representative immunofluorescence images showing the subcellular localization of phosphorylated Smad2/3 (green) in HaCaT cells, with nuclei counterstained with DAPI (blue), along with quantitative analysis of the nuclear-to-cytoplasmic (N/C) fluorescence ratio. Scale bar = 50 μm. Data are presented as mean ± SD from three independent experiments. Statistical analysis was performed using one-way ANOVA followed by Tukey’s *post-hoc* test. ***p < 0.001 vs. control; ##*p* < 0.01, ###*p* < 0.001 vs. UVB; &*p* < 0.05, &&&*p* < 0.001 vs. UVB + AzA.

As shown in Figures 6A,B, compared with the control group, UVB irradiation significantly downregulated the mRNA expression levels of the Smad-dependent target genes SERPINE1 and CTGF in HaCaT cells (*p* < 0.001). Compared with UVB irradiation alone, treatment with AzA markedly restored the expression of both SERPINE1 and CTGF in UVB-irradiated cells (*p* < 0.001). In contrast, compared with UVB-irradiated cells treated with AzA, co-treatment with the TGF-β/Smad pathway inhibitor SB431542 significantly attenuated the AzA-induced transcriptional upregulation of SERPINE1 and CTGF (*p* < 0.001), indicating that AzA-mediated transcriptional activation is dependent on intact Smad signaling.

Consistent with these transcriptional changes, immunofluorescence analysis demonstrated that, compared with the control group, UVB exposure markedly reduced the nuclear localization of phosphorylated Smad2/3 in HaCaT cells, as reflected by a significant decrease in the nuclear-to-cytoplasmic (N/C) fluorescence ratio (*p* < 0.001; Figure 6C). Compared with UVB irradiation alone, AzA treatment significantly increased p-Smad2/3 nuclear accumulation in UVB-irradiated cells (*p* < 0.01). Notably, compared with AzA treatment under UVB irradiation, co-treatment with AzA and SB431542 significantly reduced the nuclear-to-cytoplasmic (N/C) fluorescence ratio of p-Smad2/3 (*p* < 0.05), indicating that blockade of TGF-β/Smad signaling attenuates AzA-induced p-Smad2/3 nuclear translocation.

### Keratinocyte-derived conditioned medium mediates AzA-dependent regulation of fibroblast ECM metabolism via Smad signaling

**Figure 7:**
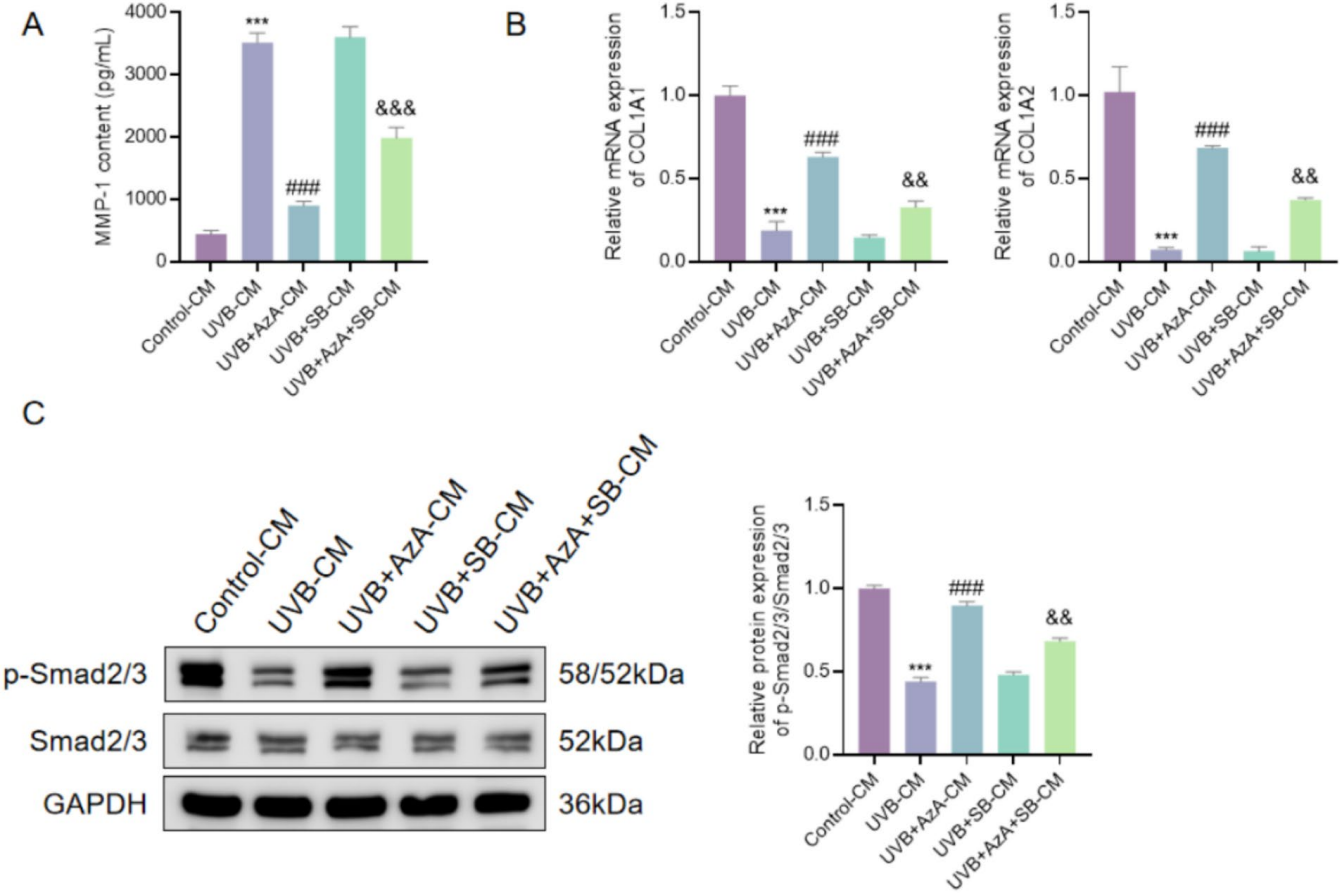
AzA regulates fibroblast ECM metabolism through keratinocyte-derived paracrine Smad signaling. **(A)** MMP-1 content in the culture supernatants of HDFs treated with conditioned medium (CM) collected from HaCaT cells under the indicated conditions. **(B)** Relative mRNA expression level of COL1A1 in HDFs treated with different conditioned media. **(C)** Relative mRNA expression level of COL1A2 in HDFs treated with different conditioned media. **(D)** Representative Western blot images and quantitative analysis of p-Smad2/3 and total Smad2/3 expression in HDFs following CM treatment. GAPDH was used as a loading control. Data are presented as mean ± SD from three independent experiments. Statistical analysis was performed using one-way ANOVA followed by Tukey’s *post-hoc* test. ***p < 0.001 vs. Control-CM; ###*p* < 0.001 vs. UVB-CM; &&*p* < 0.01, &&&*p* < 0.001 vs. UVB+AzA-CM.

As shown in Figure 7A, conditioned medium (CM), defined as culture supernatants collected from HaCaT keratinocytes subjected to the indicated treatments, derived from UVB-irradiated HaCaT cells (UVB-CM), significantly increased MMP-1 secretion in HDFs compared with control-CM (*p* < 0.001). Compared with UVB-CM, conditioned medium from AzA-treated keratinocytes (UVB+AzA-CM) markedly reduced MMP-1 levels in HDFs (*p* < 0.001). In contrast, compared with UVB+AzA-CM, co-treatment of keratinocytes with AzA and the TGF-β/Smad pathway inhibitor SB431542 significantly attenuated the inhibitory effect of AzA on MMP-1 secretion (*p* < 0.001).

Consistent with these changes in MMP-1 production, exposure of HDFs to UVB-CM markedly downregulated the mRNA expression levels of COL1A1 and COL1A2 compared with control-CM (*p* < 0.001; Figures 7B,C). Compared with UVB-CM, treatment with UVB+AzA-CM significantly restored the expression of both collagen genes in HDFs (*p* < 0.001). This restorative effect was partially abrogated when SB431542 was included during keratinocyte treatment, as evidenced by significantly reduced COL1A1 and COL1A2 expression levels compared with UVB+AzA-CM (*p* < 0.01).

Furthermore, Western blot analysis demonstrated that, compared with control-CM, UVB-CM significantly reduced the phosphorylation level of Smad2/3 in HDFs (*p* < 0.001), whereas UVB+AzA-CM markedly enhanced Smad2/3 phosphorylation relative to UVB-CM (*p* < 0.001; Figure 7D). Notably, this AzA-associated increase in Smad2/3 phosphorylation was significantly attenuated when SB431542 was applied during keratinocyte treatment (*p* < 0.01), indicating that AzA-mediated paracrine regulation of fibroblast ECM-related responses is dependent on Smad signaling.

## Discussion

In this study, we demonstrate that AzA effectively alleviates UVB-induced photoaging in HaCaT keratinocytes. AzA treatment significantly improved cell viability, reduced keratinocyte senescence, and restored ECM-related protein balance *in vitro*, as evidenced by increased pro-collagen I levels and reduced MMP-1 levels in culture supernatants. Mechanistically, AzA partially reversed UVB-induced suppression of the TGF-β/Smad signaling pathway. Pharmacological inhibition of this pathway using SB431542 largely abrogated the protective effects of AzA, supporting a critical role for Smad-dependent signaling in mediating its anti-photoaging activity. SB431542 is a well-characterized experimental inhibitor of TGF-β receptor I kinase that blocks Smad2/3 phosphorylation and is widely used to dissect Smad signaling *in vitro*. In this study, it was applied solely to verify the pathway dependence of AzA-mediated effects rather than as a therapeutic comparator. Importantly, transcriptional and subcellular analyses further revealed that AzA promotes Smad-dependent gene expression and facilitates the nuclear translocation of phosphorylated Smad2/3 under UVB-induced stress, indicating the functional restoration of downstream signaling rather than the isolated modulation of individual pathway components.

Photoaging is a cumulative process driven by UVB-induced oxidative stress, chronic inflammation, and progressive ECM degradation, ultimately resulting in wrinkle formation and loss of skin elasticity. UVB radiation penetrates the epidermis and directly damages keratinocytes and dermal fibroblasts, leading to excessive ROS generation and activation of matrix-degrading enzymes such as MMPs (26). Previous studies have established that UVB disrupts TGF-β/Smad signaling by reducing Smad2/3 phosphorylation, inducing inhibitory Smad7, and suppressing type I collagen synthesis (27). Consistent with these observations, the present study confirms UVB-induced impairment of Smad signaling and further extends existing knowledge by demonstrating that AzA not only restores Smad2/3 phosphorylation but also enhances its nuclear accumulation and transcriptional activity. These findings suggest a more integrated reactivation of the TGF-β/Smad signaling axis following UVB exposure.

AzA has long been utilized in dermatology for its antioxidant, anti-inflammatory, and antimicrobial properties, with reported effects including suppression of ROS production, inhibition of pro-inflammatory cytokine release, and modulation of keratinocyte differentiation (28). However, its potential role in photoaging has received limited mechanistic investigation. Our data indicate that AzA exerts protective effects that extend beyond non-specific antioxidation, involving the coordinated regulation of ECM metabolism and Smad-dependent transcriptional programs. This observation positions AzA as a signaling-modulatory agent capable of influencing cellular repair pathways in addition to mitigating oxidative damage.

TGF-β/Smad signaling is a key regulatory pathway governing collagen synthesis and ECM homeostasis in skin tissue. Previous studies have demonstrated that UVB irradiation disrupts this pathway by reducing Smad2/3 phosphorylation and inducing the inhibitory Smad7, thereby impairing collagen production and promoting matrix degradation (29). Consistent with this framework, our findings show that AzA treatment restored p-Smad2/3 levels, enhanced their nuclear accumulation, and reduced Smad7 expression under UVB stress conditions. These results indicate the functional reactivation of Smad-dependent transcriptional regulation rather than mere partial modulation of upstream signaling events. Collectively, the data support that AzA counteracts UVB-induced ECM imbalance through the restoration of Smad-dependent TGF-β signaling activity.

The precise molecular mechanisms by which AzA restores TGF-β/Smad signaling remain to be fully elucidated. Based on existing evidence and our findings, several testable hypotheses can be proposed. First, AzA may indirectly stabilize TGF-β receptor signaling by improving intracellular redox homeostasis. Previous studies have shown that AzA enhances mitochondrial function and scavenges ROS, thereby attenuating oxidative stress-mediated disruption of signaling cascades. Second, AzA may modulate transcription factors such as AP-1 and NF-κB, which are known antagonists of TGF-β/Smad signaling and potent inducers of MMP expression (30, 31). The suppression of these pathways could relieve inhibitory pressure on Smad signaling and favor ECM preservation. These mechanisms are not mutually exclusive and may act in concert, representing a multi-level regulatory model that warrants further experimental validation.

Notably, higher concentrations of AzA (≥30–40 mM) exhibited cytotoxic effects *in vitro*, which may reflect non-specific influences such as osmotic stress or local pH alterations rather than pathway-specific toxicity. This observation underscores the importance of considering formulation strategies, dosing optimization, and delivery systems when translating *in vitro* findings to clinical or cosmeceutical applications. Future studies employing lower-dose exposure models, sustained-release formulations, or *in vivo* systems will be essential to clarify the therapeutic range of AzA.

An additional strength of this study lies in the use of a keratinocyte–fibroblast conditioned medium model, which provides mechanistic insight into epidermal–dermal crosstalk. Our results suggest that AzA-mediated activation of Smad signaling in keratinocytes can indirectly modulate fibroblast ECM metabolism via paracrine signaling, thereby extending the relevance of our findings beyond a keratinocyte monoculture system. This approach highlights the critical role of epidermal signaling in coordinating dermal remodeling during photoaging and tissue repair processes.

Collectively, our findings support the concept that AzA functions as a dual-action anti-photoaging compound, combining antioxidant activity with regulation of endogenous repair signaling pathways. Rather than merely suppressing UVB-induced damage, AzA appears to promote intrinsic cellular repair capacity through the reactivation of TGF-β/Smad signaling and the preservation of ECM homeostasis. This paradigm aligns with emerging therapeutic strategies in skin biology that emphasize activation of endogenous repair mechanisms. In this context, a recent meta-analysis demonstrated that autologous epidermal cell suspension therapy significantly enhances skin lesion healing by stimulating intrinsic regenerative processes (32). Conceptually, these findings reinforce the value of interventions—such as AzA—that enhance the reparative potential of resident skin cells.

Reactivation of TGF-β/Smad signaling has been increasingly recognized as a promising anti-photoaging strategy. Natural polyphenolic compounds, such as resveratrol (33) and curcumin (34), have been reported to restore Smad2/3 activity and suppress MMP expression in experimental models. However, despite their mechanistic potential, these agents are often limited by poor stability, low skin permeability, variable bioavailability, and a relative lack of standardized clinical evidence in dermatological applications. In contrast, AzA is a clinically established small molecule with well-documented safety, favorable tolerability, and widely available topical formulations in dermatology. This translational readiness distinguishes AzA from many experimental anti-aging compounds and suggests that modulation of Smad-dependent signaling by AzA may represent a more readily implementable strategy in both clinical and cosmeceutical settings.

Beyond canonical signaling pathways, increasing evidence indicates that tissue repair and regeneration are tightly regulated by non-coding RNA networks. Studies on circular RNAs have demonstrated their critical roles in regulating pathways such as Wnt/β-catenin and PI3K/Akt during epithelial proliferation and migration (35, 36). Although these studies focused primarily on intestinal mucosal repair, their findings underscore a broader molecular framework that may also apply to epidermal renewal and dermal repair following photodamage. Whether AzA influences these non-coding RNA-mediated regulatory networks represents an intriguing avenue for future research.

Several limitations should be acknowledged. First, this study was confined to *in vitro* systems, including immortalized keratinocytes and a keratinocyte–fibroblast conditioned medium model; validation in *in vivo* photoaging models and human skin tissue is necessary to confirm physiological relevance. Second, the relatively high concentrations of AzA used *in vitro* may pose challenges for direct clinical translation, highlighting the need for optimized formulations to achieve effective skin exposure. Third, although Smad-dependent transcriptional activation was demonstrated, a comprehensive analysis of downstream target gene networks remains lacking. Fourth, cellular senescence was primarily assessed using SA-β-gal staining, which may not fully capture the complexity of senescence. Finally, although this study focused on Smad-dependent TGF-β signaling, UVB-induced photoaging involves additional interconnected pathways, including MAPK, p53, PI3K/Akt, and inflammatory signaling. Whether AzA modulates these parallel, stress-responsive pathways warrants further investigation.

In conclusion, AzA effectively mitigates UVB-induced photoaging by restoring Smad-dependent TGF-β signaling, preserving ECM balance, promoting Smad-mediated transcription, and reducing cellular senescence. These findings provide mechanistic insight into AzA’s protective role, particularly in the context of endogenous repair signaling and epidermal–dermal paracrine interactions *in vitro*, and support its continued investigation as a candidate therapeutic molecule for anti-photoaging and skin regenerative applications.

## Conclusion

In summary, AzA mitigates UVB-induced photoaging in keratinocytes by restoring Smad-dependent TGF-β signaling, rebalancing ECM-related protein levels, and supporting epidermal–dermal paracrine regulation under UVB stress *in vitro*. These findings position AzA not only as an antioxidant agent but also as a modulator of endogenous repair signaling under UV stress. Future studies should validate these effects in *in vivo* and clinically relevant models and further define their interaction with additional UVB-responsive signaling pathways. Moreover, optimization of topical formulation and skin delivery strategies will be essential to facilitate the translational application of AzA in anti-photoaging interventions.

## Data Availability

The original contributions presented in the study are included in the article/Supplementary material, further inquiries can be directed to the corresponding author.
