# Supplementary material for: Azelaic acid alleviates UVB-induced photoaging in keratinocytes by restoring Smad-dependent TGF-β signaling
Source: Front Med (Lausanne). 2026 Apr 7;13:1758702. doi: 10.3389/fmed.2026.1758702 (PMC13095602; doi:10.3389/fmed.2026.1758702)
Supplement: Supplementary file 1 [file Table_1.DOCX]

### ****Supplementary Table S1. Primer sequences used for RT-qPCR****

| **Gene** | **Primer** | **Sequence (5′–3′)** |
| --- | --- | --- |
| SERPINE1 | Forward | GCCATTACTACGACATCCTG |
| SERPINE1 | Reverse | AGTCAGCCTGAAACTGTCTG |
| CTGF (CCN2) | Forward | AGGGCAAAAAGTGCATCCGTA |
| CTGF (CCN2) | Reverse | TCATGCCATGTCTCCGTACAT |
| COL1A1 | Forward | CTGGTCCCAAAGGTGCTG |
| COL1A1 | Reverse | CTTTAGCACCAGCATCACCA |
| COL1A2 | Forward | CTAACCAAGGATGCACTATGG |
| COL1A2 | Reverse | GCCATTTCCTTGGAAGTCAC |
| GAPDH | Forward | AAGATCATCAGCAATGCCTCC |
| GAPDH | Reverse | AGGTTTTTCTAGACGGCAGG |
